# AutoMLST2: a web server for phylogeny and microbial taxonomy

**DOI:** 10.1093/nar/gkaf397

**Published:** 2025-05-13

**Authors:** Bita Pourmohsenin, Arthur Wiese, Nadine Ziemert

**Affiliations:** Translational Genome Mining for Natural Products, Interfaculty Institute of Microbiology and Infection Medicine Tübingen (IMIT), Interfaculty Institute for Biomedical Informatics (IBMI), University of Tübingen, Tübingen, Baden-Württemberg 72076, Germany; Translational Genome Mining for Natural Products, Interfaculty Institute of Microbiology and Infection Medicine Tübingen (IMIT), Interfaculty Institute for Biomedical Informatics (IBMI), University of Tübingen, Tübingen, Baden-Württemberg 72076, Germany; Translational Genome Mining for Natural Products, Interfaculty Institute of Microbiology and Infection Medicine Tübingen (IMIT), Interfaculty Institute for Biomedical Informatics (IBMI), University of Tübingen, Tübingen, Baden-Württemberg 72076, Germany; German Center for Infection Research (DZIF), Partner Site Tübingen, 72076, Tübingen, Germany

## Abstract

Accurate and accessible phylogenetic analysis is essential for understanding microbial taxonomy and evolution, which are integral to microbiology, ecology, and drug discovery, yet it remains a challenging task. AutoMLST2 (https://automlst2.ziemertlab.com) is a web server designed to facilitate automated phylogenetic reconstruction and microbial taxonomy analysis for bacterial and archaeal genomes. It builds on the foundation of AutoMLST, which remains widely used due to its user-friendly interface compared to similar tools. Given its continued popularity and utility, we have enhanced AutoMLST to leverage newer reference databases and computational tools. AutoMLST2 integrates the Genome Taxonomy Database, extends support to archaeal genomes, and improves analytical flexibility. Key improvements include more customizable processing modes, containerization to prevent queue accumulations, and parallel computing for large-scale studies. By incorporating up-to-date databases and workflows, AutoMLST2 continues to provide an accessible and efficient platform for researchers in microbiology, evolutionary ecology, and natural product discovery.

## Graphical Abstract

**Graphical Abstract:**
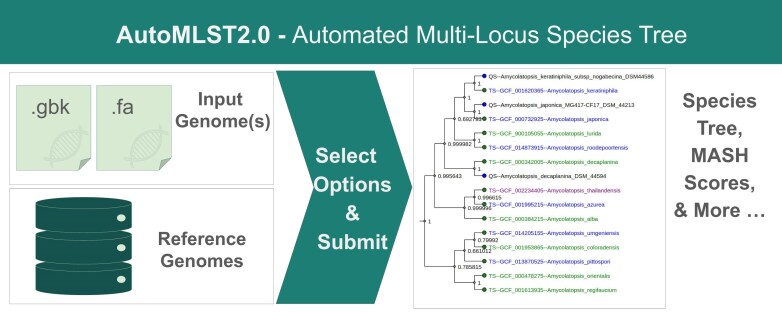

## Introduction

Understanding microbial phylogeny and taxonomy is fundamental to various biological disciplines, including microbiology, ecology, and drug discovery [1, 2]. Accurate species identification guides comparative genomic analyses and investigations of gene function and metabolic pathways [3, 4]. Traditional methods, such as 16S rRNA gene-based classification, often struggle to resolve closely related species due to their limited phylogenetic resolution [2]. Advances in whole-genome sequencing and computational methods have enabled more robust approaches, such as Average Nucleotide Identity (ANI) analysis and Multi-Locus Sequence Analysis (MLSA) [5–7]. However, implementing these workflows typically requires technical expertise and significant computational resources, making them less accessible to researchers without bioinformatics training [6, 7].

To bridge this gap, AutoMLST was developed as a user-friendly web server for automated phylogenetic analysis based on MLSA [8]. Since its release, AutoMLST has enabled rapid, high-resolution phylogenetic tree generation for bacterial species and has remained widely used due to its ease of use. However, as the field has evolved, so have the expectations for phylogenetic tools [1, 7].

Since the release of AutoMLST, GTDB-Tk has emerged as a highly accurate tool for microbial classification, leveraging the standardized Genome Taxonomy Database (GTDB) [1, 7]. However, GTDB-Tk requires substantial computational infrastructure and technical expertise, limiting its accessibility for non-expert users [6]. Despite its older database, AutoMLST remains widely used due to its user-friendly interface [8]. To address these challenges, we introduce AutoMLST2, a major update that integrates GTDB reference genomes, combining the accuracy of GTDB-Tk with the ease of use of AutoMLST. Key improvements include support for archaeal genomes, enhanced analytical flexibility, and a more scalable computational framework. By incorporating newer databases and advanced analytical capabilities, AutoMLST2 provides an accessible and efficient platform for microbial genome studies while maintaining the usability that made AutoMLST popular. Here, we outline its enhancements and demonstrate its utility in phylogenetic analysis.

## Materials and methods

### Overall workflow

**Figure 1.**
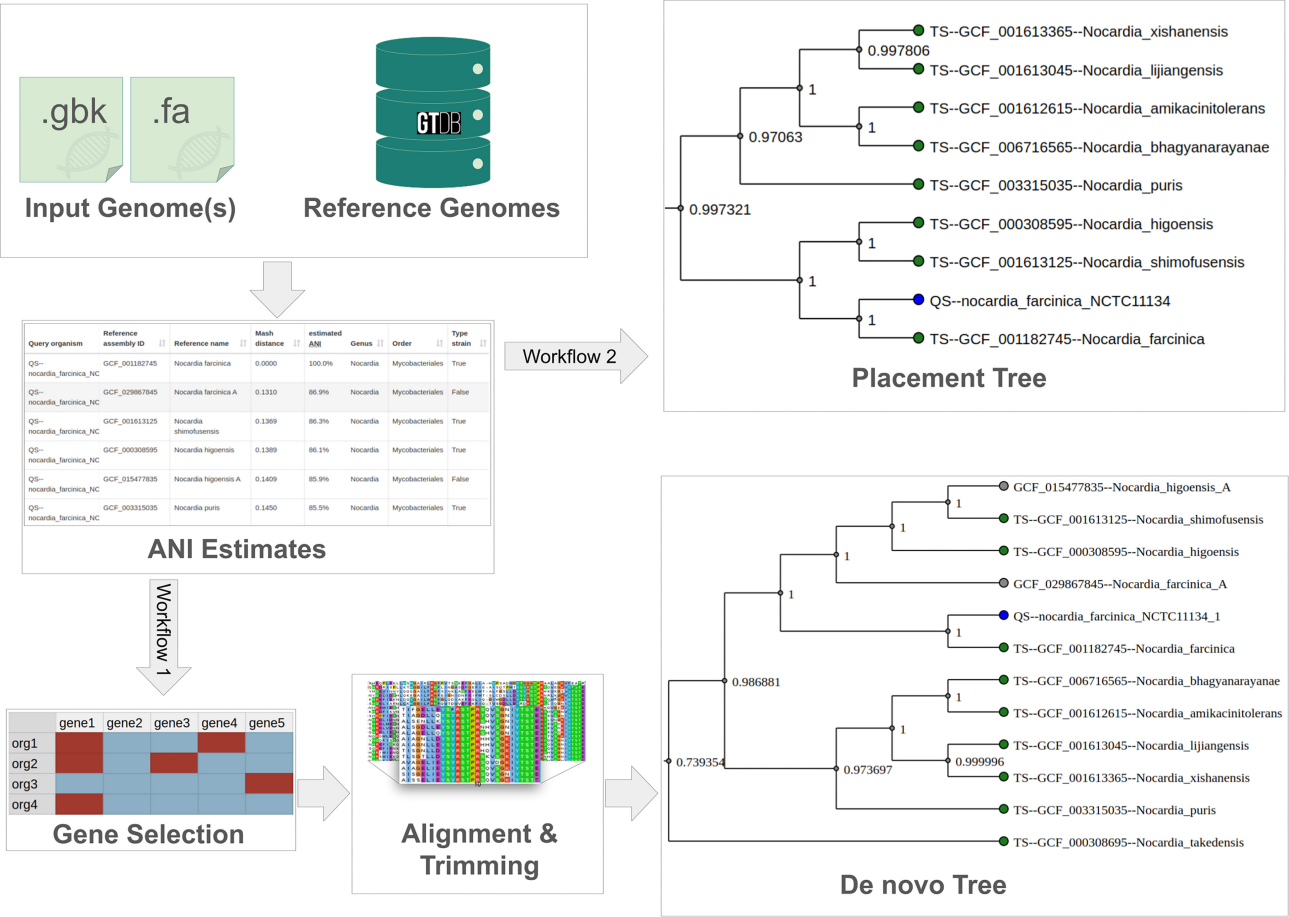
The overall workflow of AutoMLST2, automating microbial phylogenetic analysis through De novo and Placement mode workflows. Users upload genomes, which are compared to GTDB reference genomes using ANI estimation [1, 5]. Relevant genes are dynamically selected, aligned, and trimmed before phylogenetic tree construction with IQ-TREE [16] for concatenated analysis or ASTRAL-Pro3 for coalescent analysis [17]. The final De novo or Placement trees provide high-resolution evolutionary insights.

AutoMLST2 provides an automated workflow for microbial phylogenetic analysis, taking the genome(s) uploaded by the user as input (Fig. 1). Once the user selects the desired options and submits the job, AutoMLST2 compares the uploaded genomes with a reference database to identify the most similar reference genomes [1, 7]. These reference genomes are then used to construct a phylogenetic species tree, providing an evolutionary context for the query genomes [5, 9]. The platform offers two distinct analysis modes: De novo and Placement modes. In De novo mode [10, 11], phylogenetic trees are constructed entirely from scratch, while Placement mode integrates query genomes into a precomputed reference tree, which is pruned to retain only the most closely related genomes [1].

#### Input and reference selection

Users can upload up to 50 query genomes in GenBank or FASTA format. To identify appropriate reference genomes, AutoMLST2 utilizes the GTDB representative genome set (release R220, 2024), which contains ∼107 000 bacterial genomes [7]. ANI estimates are rapidly calculated using precomputed MASH sketches [12], enabling the selection of the 50 most similar reference genomes for downstream analysis.

#### Gene selection and alignment

Gene homologs are detected using >2800 filtered HMMs (Hidden Markov Models) from the PGAP (NCBI Prokaryotic Genome Annotation Pipeline) [13] database, focusing on housekeeping genes for higher resolution [14]. HMM searches are performed on protein sequences, while all alignments for phylogenetic inference are done on the corresponding nucleotide sequences. Sequences are aligned with MAFFT to ensure consistency [15].

#### Phylogenetic tree construction

AutoMLST2 supports two approaches for phylogenetic tree construction. In the concatenated approach, gene alignments are merged into a supermatrix, which is used to construct a phylogenetic tree with IQ-TREE [16]. Alternatively, in the coalescent approach, individual gene trees are inferred separately, and ASTRAL-PRO3 is employed to estimate the species tree from these independent gene trees [17].

#### Visualization and output

The final results, including phylogenetic trees and sequence alignments, are accessible through interactive visualization tools on the web interface and can be downloaded for further analysis [18].

## Results

**Figure 2.**
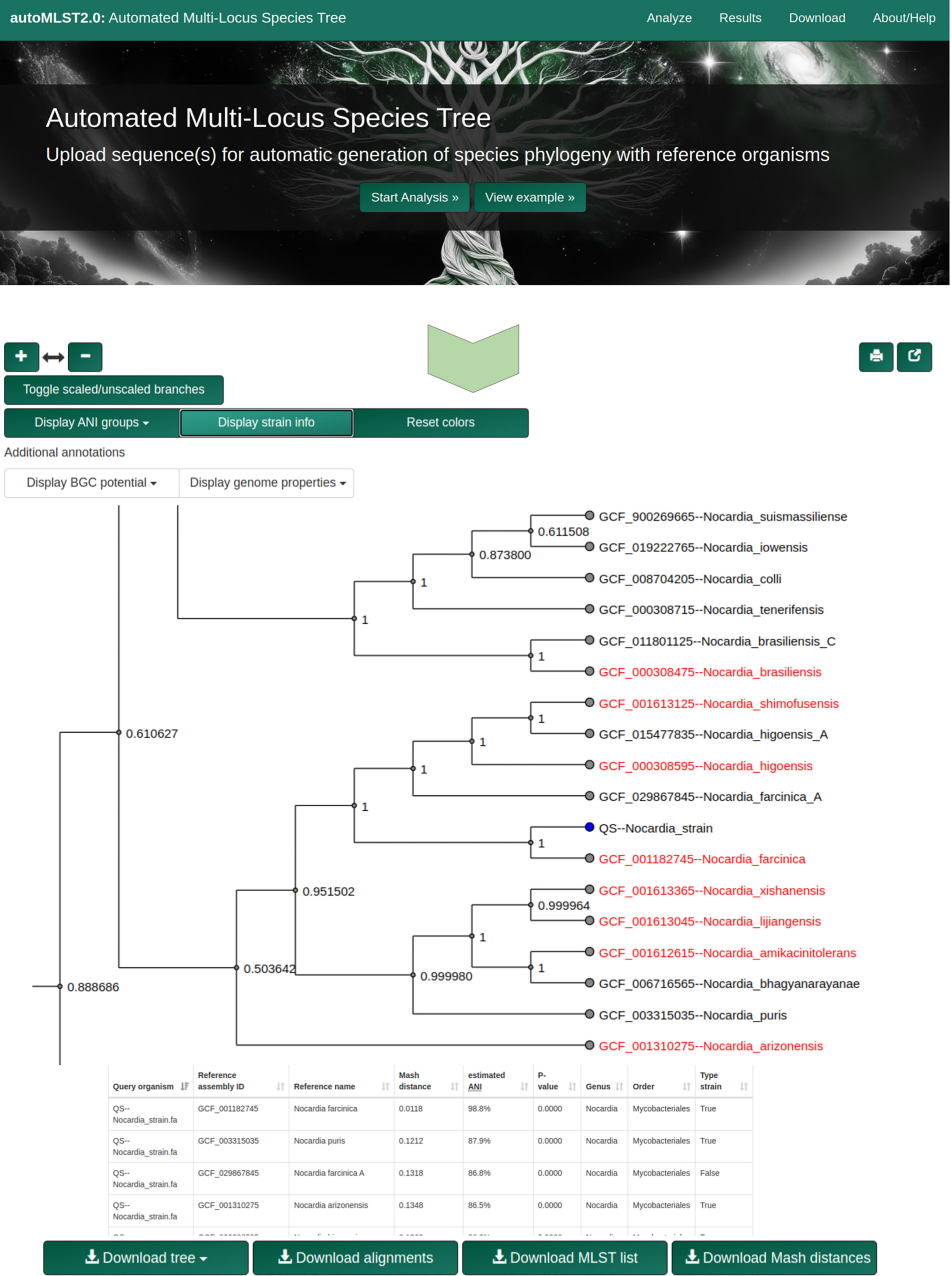
AutoMLST2 web interface, displaying a phylogenetic tree with annotated ANI groups, strain information, and genome properties. Users can visualize relationships, interact with tree elements, and download results.

AutoMLST2 is a web server designed for automated microbial phylogenetic analysis. Through its analysis page, users can upload genome files, select analysis options, and initiate processing with a single click (Fig. 2). The platform is freely available at https://automlst2.ziemertlab.com and remains open-source, ensuring accessibility for researchers of all expertise levels.

To provide a more accurate and scalable solution, AutoMLST2 incorporates several key enhancements over its predecessor. The integration of the latest GTDB release ensures that taxonomic classifications remain aligned with current genomic standards [1]. The platform also extends support for archaeal genomes, with updated HMM models improving the detection of phylogenetic signals [16]. To enhance computational efficiency, AutoMLST2 employs containerization, enabling simultaneous job processing, increasing throughput, and reducing wait times.

AutoMLST2 further improves phylogenetic accuracy, particularly for closely related genomes. Unlike GTDB-Tk, which relies on a fixed set of 120 conserved marker proteins, AutoMLST2 dynamically selects multi-locus genes from a curated set of >2800 housekeeping genes, providing higher resolution and more precise taxonomic classification. Additionally, the standalone version offers customizable workflows, allowing users to optimize analyses by selecting between fast and accurate modes.

### Benchmarking and validation

**Figure 3.**
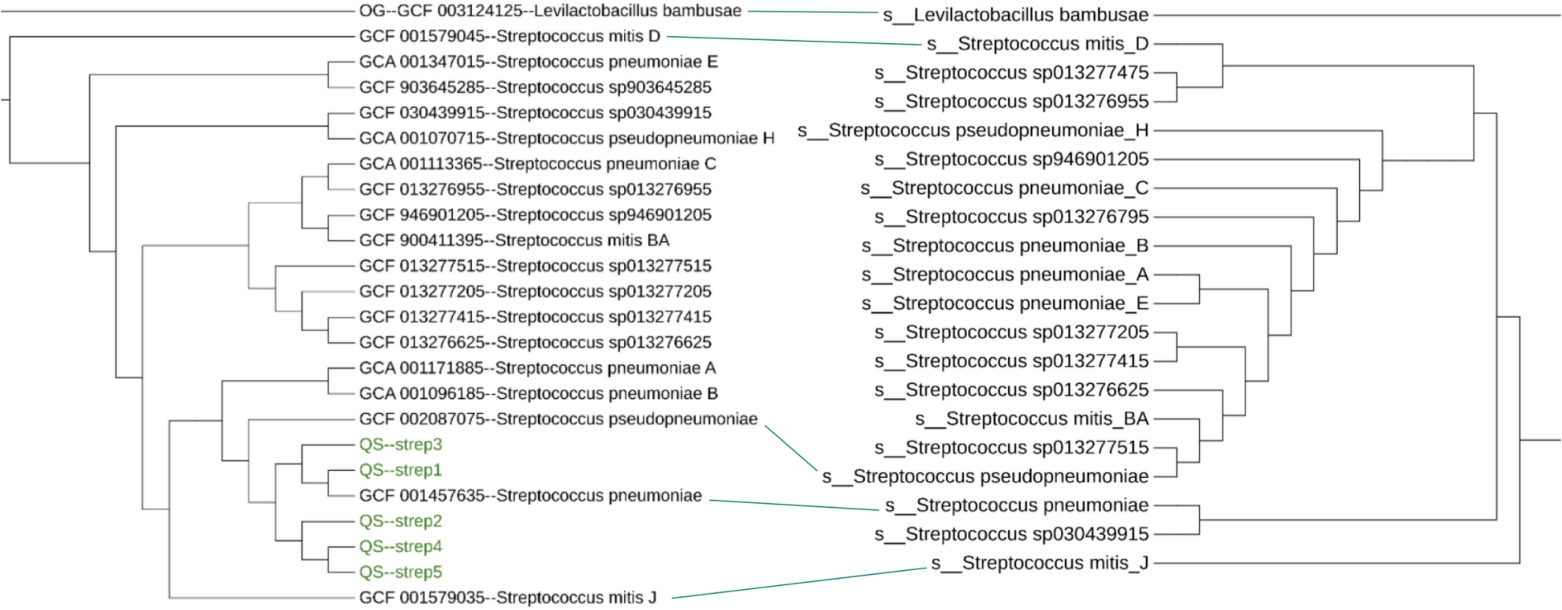
Partial tanglegram of the AutoMLST2 De novo tree for five *Streptococcus pneumoniae* strain genomes, compared to part of the GTDB reference tree. All the genomes were correctly clustered with the *S. pneumoniae* representative genome.

To evaluate AutoMLST2’s performance, we benchmarked it against the GTDB tree using a diverse set of microbial genomes. We tested its accuracy and scalability across different taxonomic levels, including closely related and distantly related genomes. Additionally, we assessed the placement mode by analyzing how well the tool integrates query genomes into existing phylogenies under various scenarios (Table 1). In terms of accuracy, AutoMLST2 performs comparably to GTDB (Fig. 3), offering more precise taxonomic classification than AutoMLST due to its incorporation of an updated reference genome database. In De novo mode, AutoMLST2 is slightly slower than AutoMLST, when analyzing a single genome, as it searches against a much larger set of HMMs. However, it scales significantly better for larger datasets, benefiting from parallel computing. Placement mode is much faster but solely depends on the ANI estimations, therefore its accuracy can decrease with the quality of genomes [especially for Metagenome-Assembled Genomes (MAGs)]. An extra set of four example analyses demonstrating AutoMLST2’s performance across different scenarios, including isolates and MAGs, is available at https://automlst2.ziemertlab.com/results/example1 up to https://automlst2.ziemertlab.com/results/example5 as well as in the supplementary data.

**Table 1.** Taxonomic precision of genome placement across different datasets and tree-building methods

Dataset/scenario	Taxonomic precision
Multiple sets of reference genomes	100% of genome taxonomic assignments match GTDB
10 random isolate genomes from Actinobacteriota	100% of genome genus assignments match GTDB
10 random Gammaproteobacteria genomes, a mix of MAGs and isolates	2 low-quality MAGs are not present in the final tree. Other genomes match GTDB.
*Nocardia farcinica* NCTC 11134 Placement tree	Correct Placement into the GTDB tree
*Nocardia farcinica* NCTC 11134 Coalescent tree	Query assignment matches GTDB, final tree slightly different from GTDB
*Nocardia farcinica* NCTC 11134 Concatenated tree	Query assignment matches GTDB, final tree slightly different from GTDB
*Cyanobacterium sp002813895* (randomly chosen)	Taxonomic assignment matches GTDB

Genome assignments were compared against the GTDB, with high accuracy observed for reference genomes and isolate genomes. Low-quality MAGs were sometimes excluded from the final tree. *Nocardia farcinica* NCTC 11134 placement was consistent across different tree-building approaches, with slight variations in final topology.

## Discussion

AutoMLST2 advances automated microbial phylogenetic analysis by integrating modern genomic resources and addressing limitations of its predecessor. By incorporating the GTDB and supporting archaeal genomes, AutoMLST2 ensures taxonomic classifications remain up to date with current genomic standards [1]. Its dual-mode workflow—offering both *de novo* phylogenetic reconstruction and genome placement—enhances adaptability across diverse microbial studies [10,11].

A key strength of AutoMLST2 is its user-friendly web interface, which simplifies complex analyses for researchers of all bioinformatics backgrounds [8]. Unlike command-line tools, it provides interactive visualizations, facilitating intuitive exploration of phylogenetic relationships. Automated reference genome selection and dynamic gene filtering improve resolution, particularly for closely related microbial strains, where fixed-marker approaches may be less effective [5,7].

In benchmarking, AutoMLST2 demonstrated high taxonomic precision across diverse datasets. Notably, in the case of *N. farcinica* NCTC 11134, the coalescent tree differed slightly from the GTDB reference tree. This variation likely reflects AutoMLST2’s use of a dynamic set of conserved genes, which can provide a higher phylogenetic signal for closely related genomes.

AutoMLST2 efficiently processes large datasets, making it valuable for microbiology, evolutionary biology, and natural product discovery. While it performs well with high-quality MAGs, its accuracy decreases with lower-quality assemblies due to fragmented genomes limiting phylogenetic inference. Future improvements could focus on optimizing gene selection for fragmented datasets and integrating network-based analyses of biosynthetic diversity.

AutoMLST2 is publicly available at https://automlst2.ziemertlab.com, with comprehensive documentation for users at all expertise levels. Its combination of accuracy, flexibility, ease of use, and advanced visualization tools makes it a powerful resource for microbial phylogenetics.

## Supplementary Material

gkaf397_Supplemental_Files

## Data Availability

All data generated and analyzed in this study are publicly available. Phylogenetic trees, benchmarking results, and additional supporting datasets, including example analyses and workflow results, are provided in the supplementary data. Genome data can be accessed through NCBI GenBank, with accession numbers listed in the trees. The AutoMLST2 code and reference datasets are available at https://automlst2.ziemertlab.com/download.
